# Pan-Filovirus Serum Neutralizing Antibodies in a Subset of Congolese Ebolavirus Infection Survivors

**DOI:** 10.1093/infdis/jiy453

**Published:** 2018-08-13

**Authors:** Matthew S Bramble, Nicole Hoff, Pavlo Gilchuk, Patrick Mukadi, Kai Lu, Reena H Doshi, Imke Steffen, Bradly P Nicholson, Allen Lipson, Neerja Vashist, Cyrus Sinai, D’andre Spencer, Garrard Olinger, Emile Okitolonda Wemakoy, Benoit Kebela Illunga, James Pettitt, James Logue, Jonathan Marchand, Justin Varughese, Richard S Bennett, Peter Jahrling, Guy Cavet, Tito Serafini, Erica Ollmann Saphire, Eric Vilain, Jean Jacques Muyembe-Tamfum, Lisa E Hensely, Graham Simmons, James E Crowe, Anne W Rimoin

**Affiliations:** 1 Department of Epidemiology, School of Public Health, University of California, Los Angeles; 2 Department of Genetic Medicine Research, Children’s Research Institute, Children’s National Medical Center, Washington, District of Columbia; 3 Vanderbilt Vaccine Center, and Department of Pathology, Microbiology, and Immunology, Vanderbilt University Medical Center, Nashville, Tennessee; 4 Institut National de Recherche Biomedicale, Kinshasa, Democratic Republic of the Congo; 5 Blood Systems Research Institute, and Department of Laboratory Medicine, University of California, San Francisco; 6 Institute for Medical Research, Durham Veterans Affairs Medical Center, North Carolina; 7 Boston University, School of Medicine, Department of Medicine, Massachusetts; 8 Kinshasa School of Public Health, Kinshasa, Democratic Republic of the Congo; 9 Direction de la Lutte Contre les Maladies, Ministère de la Sante, Kinshasa, Democratic Republic of the Congo; 10 Integrated Research Facility at Fort Detrick, National Institute of Allergy and Infectious Diseases (NIAID), National Institutes of Health (NIH), Frederick, Maryland; 11 Atreca, Inc, Redwood City; 12 Skaggs Institute for Chemical Biology, La Jolla, California; 13 Department of Immunology and Microbial Science, Scripps Research Institute, La Jolla, California; 14 Emerging Viral Pathogens Section, NIAID, NIH, Frederick, Maryland; 15 Departments of Pediatrics and Pathology, Microbiology, and Immunology, Vanderbilt University Medical Center, Nashville, Tennessee

**Keywords:** Ebola, DRC, filovirus, immune response

## Abstract

One year after a Zaire ebolavirus (EBOV) outbreak occurred in the Boende Health Zone of the Democratic Republic of the Congo during 2014, we sought to determine the breadth of immune response against diverse filoviruses including EBOV, Bundibugyo (BDBV), Sudan (SUDV), and Marburg (MARV) viruses. After assessing the 15 survivors, 5 individuals demonstrated some degree of reactivity to multiple ebolavirus species and, in some instances, Marburg virus. All 5 of these survivors had immunoreactivity to EBOV glycoprotein (GP) and EBOV VP40, and 4 had reactivity to EBOV nucleoprotein (NP). Three of these survivors showed serologic responses to the 3 species of ebolavirus GPs tested (EBOV, BDBV, SUDV). All 5 samples also exhibited ability to neutralize EBOV using live virus, in a plaque reduction neutralization test. Remarkably, 3 of these EBOV survivors had plasma antibody responses to MARV GP. In pseudovirus neutralization assays, serum antibodies from a subset of these survivors also neutralized EBOV, BDBV, SUDV, and Taï Forest virus as well as MARV. Collectively, these findings suggest that some survivors of naturally acquired ebolavirus infection mount not only a pan-ebolavirus response, but also in less frequent cases, a pan-filovirus neutralizing response.

## Teaser

Infection with Zaire ebolavirus (EBOV) appears to trigger an anti–pan-filovirus response in a small subset of Congolese Ebola virus disease survivors, generating potential neutralizing antibodies against EBOV and Bundibugyo, Taï Forest, Sudan, and Marburg viruses.

Ebolavirus (EBOV), a member of the Filoviridae family, first discovered in 1976 [1], is a highly lethal hemorrhagic fever virus that has been responsible for thousands of deaths worldwide [2]. The filovirus family consists of 1 species of *Marburgvirus* (MARV), along with the genus *Ebolavirus*, which harbors 5 distinct species, *Zaire ebolavirus* (EBOV), *Sudan ebolavirus* (SUDV), *Taï Forest ebolavirus* (TAFV), *Reston ebolavirus*, and *Bundibugyo ebolavirus* (BDBV) [3]. While considerable work has been conducted on understanding the pathogenesis of EBOV, there is still a lack of approved vaccine therapies or treatments [4]. The majority of vaccine and therapeutic research has focused on the membrane glycoprotein (GP) envelope of the virus, which is responsible for entry of host cells and the target of neutralizing antibodies. Studies of vaccine candidates in development have shown that protection can be achieved in nonhuman primates (NHPs) against live EBOV challenge using chimeric vesicular stomatitis virus–based approaches [5–9]. In addition to experimental EBOV vaccination, successful protection against other ebolavirus species and additional members of the Filoviridae family also has been induced by vaccination in NHPs [10]. The sequences of filovirus GPs and other filoviral proteins exhibit a moderate level of conservation; therefore, it is of interest to determine if a pan-filovirus vaccination approach could be used to achieve broad protection against diverse species of filoviruses.

It is unknown if naturally occurring ebolavirus infection or vaccination can induce potent pan-filovirus polyclonal antibody responses. Development of pan-ebolavirus or pan-filovirus therapies is desirable, but most studies to date have focused only on the ebolavirus species. Cross-reactive monoclonal antibodies (mAbs) against 4 species of ebolavirus GP have been isolated from mice [11]. Two human mAbs isolated from an EBOV survivor of infection during the 2014 West African outbreak showed cross-reactive binding and neutralization of several ebolavirus species, but not MARV [12]. Here, we present evidence for a pan-filovirus polyclonal antibody response in a subset of Congolese Ebola virus disease (EVD) survivors 1 year after initial infection, during the 2014 outbreak in Boende, located in the equator province of the Democratic Republic of the Congo (DRC) [13]. Using several immunological measures, we determined that a subset of 15 confirmed/suspected survivors had polyclonal antibodies in serum and plasma that reacted to the GP proteins of EBOV, BDBV, TAFV, SUDV, and MARV. Pseudovirus neutralization and entry inhibition assays demonstrated that serum antibodies from a subset of survivors neutralized EBOV, SUDV, BDBV, TAFV, and MARV.

## METHODS

### Study Population

In November 2015, 15 survivors from the 2014 Boende EVD outbreak were identified using DRC Ministry of Health reports. Ethical approval was obtained at the University of California, Los Angeles Fielding School of Public Health and the Kinshasa School of Public Health. Survivors had confirmed certificates that they were EVD-free by plasma polymerase chain reaction (PCR) testing for virus genome and were being released from an Ebola Treatment Center (ETC). Six of the identified participants were considered confirmed cases with a positive PCR on entry to an ETC, and the remaining participants were suspected cases based on Ministry of Health reports along with in-person interviews, and confirmation from healthcare workers present during the outbreak.

### Sample Collection

Weight, height, and blood pressure were measured, and blood specimens were obtained from participants by venipuncture in Vacutainer tubes (BD Biosciences). After processing, aliquots of serum, plasma, and lymphocytes from buffy coat preparations were frozen and stored in a liquid nitrogen dry shipper at the Institut National de Recherché Biomedicale in Kinshasa and shipped to collaborating institutions as previously described [14].

### Enzyme-Linked Immunosorbent Assays

We measured plasma antibodies against EBOV, BDBV, SUDV, or MARV GP by enzyme-linked immunosorbent assay (ELISA). Microtiter plate wells were coated with purified, recombinant ectodomain of 1 of the 4 recombinant GPs and incubated at 4°C overnight. Plates were blocked with 2% nonfat dry milk and 2% normal goat serum in Dulbecco’s phosphate-buffered saline containing 0.05% Tween-20 for 1 hour. Serial 3-fold dilutions of plasma or GP-specific immunoaffinity-purified polyclonal antibodies in blocking buffer were added in triplicate to the wells and incubated for 1 hour at ambient temperature. Nonimmune plasma served as a control for binding specificity. Bound antibodies were detected using goat antihuman immunoglobulin G (IgG) conjugated with horseradish peroxidase (HRP) (Southern Biotech) and 3,3′,5,5′-tetramethylbenzidine (TMB) substrate (ThermoFisher). Color development was monitored, then 1 N hydrochloric acid was added to stop the reaction, and the absorbance was measured at 450 nm using a spectrophotometer (Biotek). A nonlinear regression analysis was performed on the resulting curves using Prism version 7 (GraphPad) to calculate the plasma dilution that yielded a half-maximum optical density (OD_450_) value. Antibody titer was expressed as the inverse of plasma dilution [15]. We also measured EBOV GP and nucleoprotein (NP) IgG titers with a commercially available ELISA kit (Alpha Diagnostic International) following the manufacturer’s protocol.

### Luciferase Immunoprecipitation System

The C-terminal domain of Mayinga EBOV VP40 (bp 583–981) fused to Renilla luciferase antigen was expressed in COS-1 cells and used for luciferase immunoprecipitation system, as previously described [14]. VP40 antibody positivity was determined if the relative luciferase signal (S) postimmunoprecipitation was greater than the cutoff (C), as determined from an average of 8 previously identified negative serum samples plus 3 standard deviations. S/C values >1 were considered positive. Positive controls were obtained from human convalescent patient sera collected during the 2014 West African outbreak of EBOV infection [16].

### Pseudotype Virus Neutralization Assay

Pseudotype viruses were generated as described previously, using expression vectors for the GPs of EBOV, SUDV, TAFV, MARV, Lassa virus [17], or BDBV [18], and the pNL-Luc viral backbone obtained from the National Institutes of Health AIDS Reagent Program, Division of AIDS, National Institute of Allergy and Infectious Diseases (catalogue number 3418) [19]. Detailed methodology has been previously described for the pseudotyped neutralization assays [14]. All inoculations were performed in duplicate wells, and each plate contained identical controls, including uninfected cells, cells inoculated in the absence of serum, and cells inoculated in the presence of negative control serum (US donor) or positive control serum from a recent, confirmed EVD survivor [18]. Infection in the presence of human serum samples was expressed as the percentage of the signal from infection in presence of negative control serum. We determined that neutralizing activity was present when serum from the patients reduced signal to ≤50% of that from the appropriate control.

### Virus-like Particle Entry Inhibition Assay

EBOV and BDBV virus-like particles (VLPs) were produced using plasmids encoding VP40–G-LucN and EBOV GP or BDBV GP as previously described [20] and used in a G-Luc complementation assay for inhibition of viral entry. VLPs were incubated with serial dilutions of sera (1:50, 1:250, 1:1250, or 1:6250) before spin-infection of target cells (RD cells transiently expressing G-Luc), in duplicate. G-Luc activity in cell lysates was measured using a commercial kit (Pierce) according to the manufacturer’s instructions and expressed as percentage of the luciferase activity that was detected in the presence of negative control serum.

### Plaque Reduction Neutralizing Test

Neutralizing activity was measured with a plaque reduction neutralization test for 50% or 80% reduction in which live virus was incubated in the presence of serial dilutions of test samples under Biosafety Level 4 containment conditions, as previously described [14].

### GP Expression and Purification

The ectodomains of EBOV GP (residues 1–636; strain Makona; GenBank Accession KM233070), BDBV GP (residues 1–643; strain 200706291 Uganda; GenBank Accession NC_014373), SUDV GP (residues 1–637, strain Gulu; GenBank Accession NC_006432), and MARV GP aa 1–648 (strain Angola2005; GenBank Accession DQ447653) were expressed transiently in Expi293F cells with a C-terminus strep II tag, and purified using 5 mL StrepTrap HP column (GE Healthcare). GP ectodomains were purified further with Superdex200 (GE Healthcare) size exclusion chromatography (SEC) in phosphate-buffered saline (PBS). For affinity resin preparation and purification of GP-reactive polyclonal antibodies, we used EBOV GP that was produced in *Drosophila* Schneider 2 (S2) cells. In brief, complementary DNA encoding a recombinant ectodomain of EBOV GP in a modified pMTpuro vector was transfected into S2 cells followed by stable selection of transfected cells with 6 μg/mL puromycin. GP ectodomain expression was induced with 0.5 mM copper sulfate for 4 days. Protein was purified using Strep-Tactin resin (Qiagen) using an engineered strep II tag, and purified further by Superdex 200 (S200) SEC.

### Purification of GP-Reactive Polyclonal Antibodies From Plasma

Purified EBOV GP was biotinylated at a 1:20 molar ratio in PBS using EZ-Link NHS-PEG^4^-Biotin (ThermoScientific), and then buffer-exchanged into PBS with a 0.5-mL Zeba spin column (ThermoScientific) and coupled to streptavidin sepharose (GE Healthcare) at 1 mg/mL to prepare the affinity resin. Resin was washed 2 times with PBS containing 0.5 M sodium chloride, loaded with PBS, and used for affinity purification of GP-reactive antibodies as follows. Plasma was diluted 2 times in PBS, filtered through a 0.2-μm filter and applied to the resin. Unbound proteins were washed with 20 resin volumes of PBS followed by 10 resin volumes of 0.1 M glycine-HCI at pH 3.5. Bound antibodies were eluted with 5 resin volumes of 0.1 M glycine-HCI at pH 1.8 and neutralized with 1M Tris buffer to adjust the pH to 7.4. The GP-reactive IgG fraction was purified further by protein G agarose (Pierce). Purified antibodies were buffer-exchanged into PBS, concentrated, quantified, and assessed in ELISA for reactivity against EBOV, BDBV, SUDV, or MARV GPs, as detailed above. Binding to streptavidin and H1N1 influenza virus hemagglutinin protein prepared similarly to the filovirus GP served as control for binding specificity of purified antibodies in ELISA.

### Cell Surface–Displayed GP Antibody Competition-Binding Assay

Jurkat cells expressing the MARV (Angola) GP (kindly provided by Carl Davis and Rafi Ahmed) were primed proteolytically with 0.5 mg/mL thermolysin (Pierce) in PBS for 5 minutes at 34°C, then washed with PBS containing 2% of fetal bovine serum and 2 mM ethylenediaminetetraacetic acid (pH 8.0). For staining, cells were incubated for 30 minutes at 4°C with serial 2-fold dilutions of plasma in triplicate, followed by incubation for 30 minutes with 5 µg/mL fluorescently labeled MARV GP-specific mAbs. Cells were washed, fixed with 4% paraformaldehyde in PBS, and analyzed using a high-throughput flow cytometer (iQue, Intellicyt). Data were analyzed with ForeCyt software (Intellicyt). Plasma from a donor without an exposure history to filovirus infection was used as a negative control. Background values were determined from binding of second labeled antibody to untransfected Jurkat cells. Results were expressed as the percent of MARV GP-reactive antibody binding in the presence of plasma relative to a MARV-specific mAb-only control (maximal binding), minus background. For fluorescent labeling of antibodies, we used Alexa Fluor 667 NHS ester (ThermoFisher) and followed the manufacturer’s protocol. Labeled mAbs were purified further and buffer exchanged into PBS using desalting Zeba columns (ThermoFisher), and stored at 4°C.

## RESULTS

### Donor Reactivity Against Recombinant EBOV Proteins

**Figure 1.**
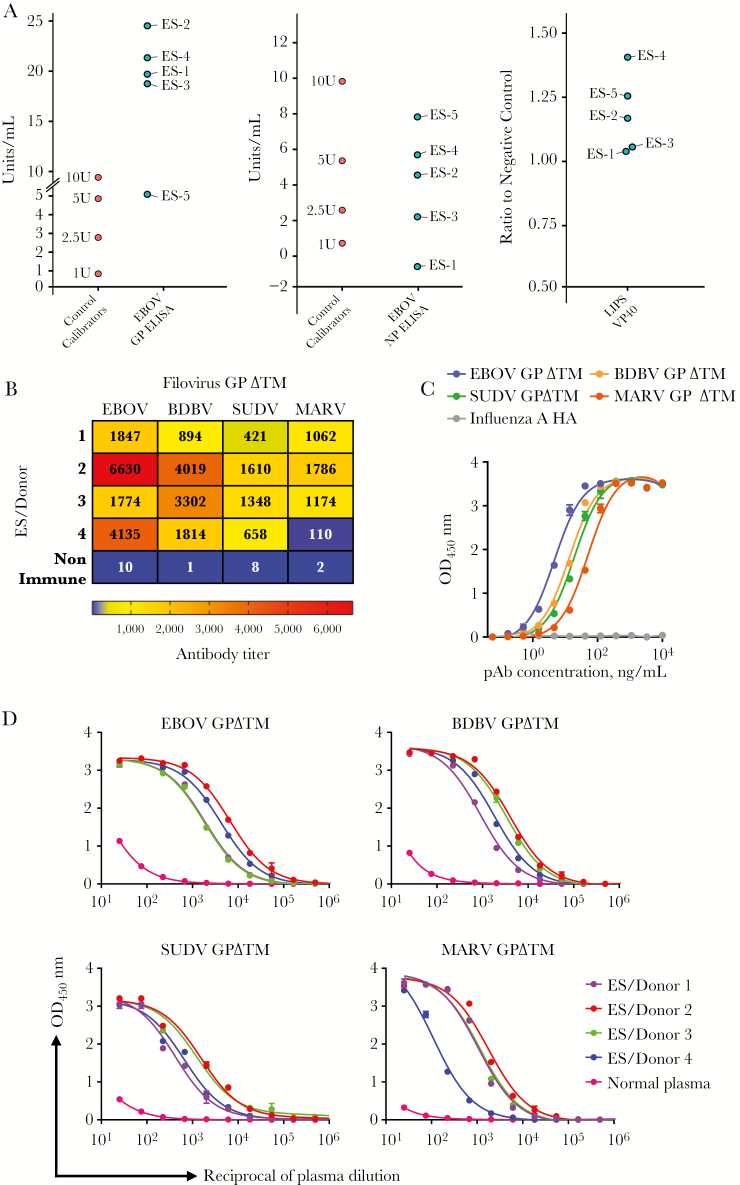
*A*, Serological assessment of donor reactivity against Zaire ebolavirus (EBOV) glycoprotein (GP) and nucleoprotein using a commercially available enzyme-linked immunosorbent assay and VP40 reactivity assessment using a luciferase immunoprecipitation assay. *B*, Reactivity of plasma antibodies to recombinant GP of ebolavirus (EBOV), Bundibugyo ebolavirus, Sudan ebolavirus, or Marburg ebolavirus, antibody titers for 4 assessed plasma specimens, which demonstrated multiple filovirus neutralization ability. *C*, Reactivity of polyclonal antibodies that were affinity purified from plasma of the subject with the most broadly reactive antibodies, subject ES 2. Data in *B–D* represent the mean ± standard deviation of technical triplicates. *D*, Representative binding curves for the 4 plasma specimens with the broadest GP reactivity profiles. Abbreviations: BDBV, Bundibugyo ebolavirus; EBOV, Ebola virus; ELISA, enzyme-linked immunosorbent assay; HA, hemagglutinin; LIPS, luciferase immunoprecipitation assay; MARV, Marburg virus; NP, nucleoprotein; OD_450_, optical density 450 nanometers; pAb, polyclonal antibody; SUDV, Sudan ebolavirus; ZEBOV, Zaire ebolavirus.

First, we measured serum antibodies to EBOV GP or NP by ELISA and focused on those individuals who demonstrated a pan-ebolavirus or pan-filovirus response in downstream neutralization assays. Of the individuals in the survivor cohort who demonstrated multiple virus neutralization capacity, all had high antibody titers against EBOV GP (>5 U/mL) (Figure 1A) and all but 1 had moderate reactivity against EBOV NP (>1.0/mL) (Figure 1A). Serum testing for EBOV VP40 in a luciferase immunoprecipitation assay showed again that all 5 of survivors that mounted a pan-ebolavirus/filovirus response retained antibodies against the viral matrix protein of EBOV 1 year after initial infection (Figure 1A). While some of the 10 additional survivors showed varying degrees of serological response to tested EBOV proteins, these individuals did not necessarily respond or neutralize in a pan-ebolavirus/filovirus manner during downstream assays (Supplementary Figure 1). These findings indicate that the survivors within this cohort who demonstrated multiple filovirus neutralization capacity exhibited some degree of reactivity to at least 2 of the EBOV recombinant proteins tested, with reactivity to EBOV GP being the most dramatic.

### Assessing Plasma Antibody Reactivity to GP of Different Filovirus Species

GP is the only known filovirus target for neutralizing antibodies, but it has a relatively low level of amino acid sequence homology between different filovirus species. Therefore, to estimate the breadth of seroreactivity for diverse species in survivors of natural EBOV infection, we assessed binding of plasma antibodies from those individuals who demonstrated pan-ebolavirus/filovirus neutralization to recombinant GP of 4 clinically relevant filovirus species—EBOV, BDBV, SUDV, and MARV. Surprisingly, plasma antibodies from these 4 donors (ES 1, 2, 3, and 4) showed variable levels of binding to GP for all 3 ebolavirus species tested, but most interestingly, 3 of these survivors displayed strong antibody binding to MARV GP as well (Figure 1B and 1D). Unfortunately, plasma quantity from donor 5 was insufficient to conduct these assays, despite what appears to be a broad ebolavirus response in other approaches. This cross-reactive binding pattern of polyclonal antibodies suggests that a single-species EBOV infection elicited serum antibodies with pan-filovirus binding capacity. Although less likely, we considered the alternative possibility that these donors might have had an additional subclinical history of infection with a second filovirus species, which resulted in development of independent, nonoverlapping virus GP-specific antibody responses. To further investigate this matter, we obtained a fraction of EBOV GP–reactive polyclonal antibodies by affinity purification from the plasma specimen with the highest titer of broadly reactive antibodies (donor ES 2). Then, we assessed binding of EBOV GP-purified antibodies to EBOV, BDBV, SUDV, or MARV GP. EBOV GP–purified antibodies exhibited broad and high reactivity to all 4 GPs, similar to the binding pattern of the plasma sample (Figure 1C). The activity was specific to GP, since the GP-purified antibodies did not react with a similarly prepared recombinant influenza hemagglutinin protein. Together, these results suggest that infection with 1 species of ebolavirus can elicit a broadly reactive antibody response to the GP of several filovirus species, including MARV, in some individuals.

### Assessing Broad Filovirus Neutralization Capacity

**Figure 2.**
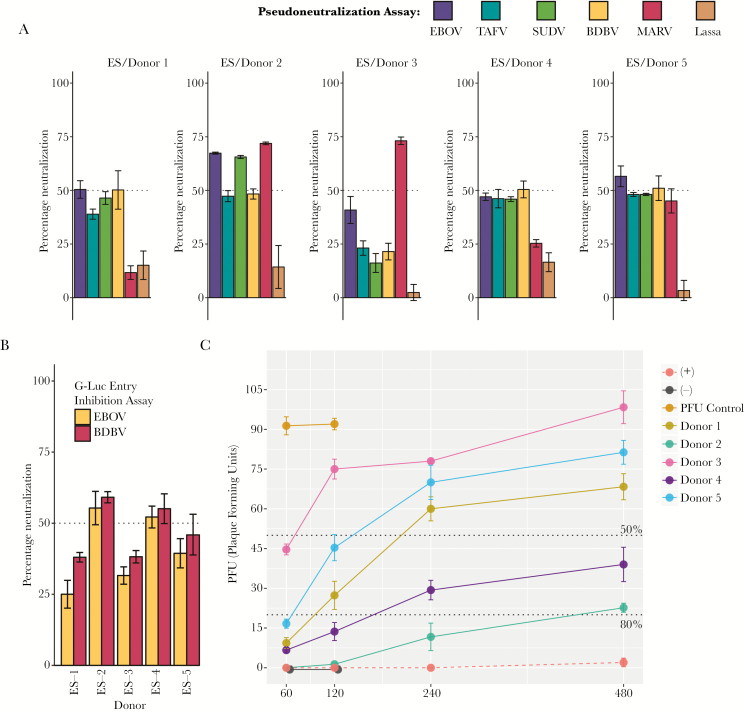
*A*, Ebolavirus species (ES) donor serum neutralizing activity against Zaire ebolavirus (EBOV), Taï Forest ebolavirus (TAFV), Sudan ebolavirus (SUDV), Bundibugyo ebolavirus (BDBV), or Marburg virus (MARV) or the arenavirus Lassa virus, using a vesicular stomatitis virus pseudovirus assay expressing a species-specific glycoprotein. Neutralization was assessed in technical triplicate using patient serum at a 1:50 dilution. *B*, Neutralizing activity in donor serum for EBOV or BDBV using the G-Luc entry inhibition assay. Neutralization was assessed in technical triplicate using patient serum at a 1:50 dilution. *C*, Plaque reduction neutralization antibody assay using live EBOV, measured in triplicate for each dilution. Results shown in colors indicate the ability to reduce EBOV plaque-forming units by at least 50% for those individuals that demonstrated a multiple filovirus neutralization pattern.

To identify that these 5 survivors from the 2014 Boende outbreak cohort had serum antibodies that were able to neutralize in a pan-ebolavirus or pan-filovirus manner, we used a recombinant vesicular stomatitis virus pseudotyped with filovirus GPs and an entry inhibition assay for EBOV and BDBV. We identified that all but 1 of the 5 donors exhibited the ability to neutralize EBOV >50% in 1 or both assays (Figure 2A and 2B). Additionally, donors 1, 2, 4, and 5 were able to neutralize live EBOV >80% and donor 3, >50% at a serum dilution of 1:60 using the gold standard plaque reduction neutralization test (Figure 2C). Interestingly, we determined that these donors were also able to neutralize other ebolavirus species to various degrees, with donor 2 demonstrating the broadest response, effectively neutralizing TAFV, SUDV, and BDBV in >50% of control using either pseudovirus assays (Figure 2A) or the entry inhibition assay (Figure 2B). Most striking, however, was the neutralizing responses that we observed against not ebolavirus, but MARV, with donors 2 and 3 exhibiting strong serum antibody neutralization capacity, >70% of control (Figure 2A). Collectively, these findings from a subset of EBOV survivors from the 2014 Boende outbreak reveal that infection with 1 strain of ebolavirus can elicit a neutralizing antibody response across the Filoviridae family.

### Binding of EBOV-Immune Plasma Antibodies to an Antigenic Site of Vulnerability on MARV GP

**Figure 3.**
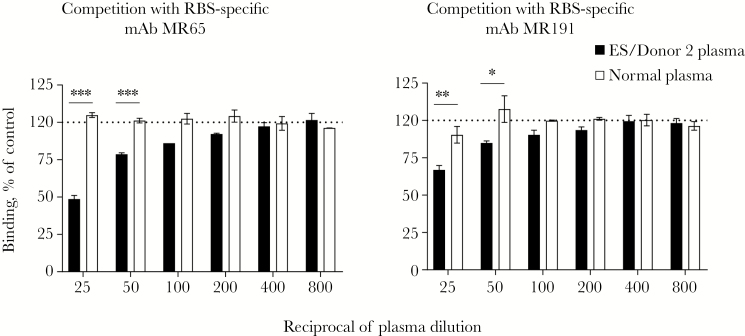
Competition-binding assay with plasma antibodies and receptor binding site (RBS)–specific monoclonal antibodies (mAbs) (5 µg/mL) using cell surface–displayed Marburg virus (MARV) glycoprotein (GP). Subject ebolavirus species (ES) 2 plasma revealed the capacity to significantly reduce binding of RBS-specific mAbs to Jurkat-MARV GP at 1:25 and 1:50 dilutions, whereas normal plasma did not. Mean ± standard deviation of assay triplicates. Dashed line indicates MARV-specific mAb-only control and shows maximal binding to MARV GP. Background was determined from binding of MARV-specific mAb to an untransfected cell line that does not express GP. Results are expressed as the percentage of MARV-specific antibody binding in the presence of plasma relative to MARV-specific mAb-only control minus background.

MARV is antigenically distinct from ebolaviruses [21]. We investigated if plasma antibodies responses from the specimen with the broadest neutralizing activity (subject ES 2) could target a known antigenic site of vulnerability on MARV GP. We used a Jurkat cell surface display method for MARV GP and a high-throughput flow cytometric assay to assess if plasma antibodies could compete with binding of 2 potent MARV mAbs previously isolated from a MARV survivor, MR65 or MR191. Antibodies MR65 and MR191 bind specifically to the receptor binding site (RBS) of MARV GP, and showed high protective capacity in animal models of MARV infection, including postexposure protection of NHPs [21, 22]. MR191 is considered as a lead therapeutic antibody candidate for postexposure treatment of MARV infection [22]. At the lowest plasma dilution tested (1:25), polyclonal antibodies reduced binding of mAb MR65 or MR191 by 51% or 37%, respectively. Normal plasma did not exhibit blocking activity when compared to a no-plasma control (Figure 3). This finding suggested that at least a fraction of potent pan-filovirus GP-reactive antibodies in this donor are directed against a vulnerable site on the most antigenically distinct GP, that of MARV.

## DISCUSSION

The breadth of human antibody responses to filoviruses has been a subject of interest in recent studies. Previously it was thought that it would be difficult to identify antibodies that neutralize >1 species of filovirus because of the antigenic diversity in GP from different strains [23]. Recently, human mAbs were described that neutralize 2 species of ebolaviruses. Here, we demonstrate that some survivors of the 2014 Boende, DRC outbreak of EBOV have serum neutralizing antibodies against all tested species of ebolavirus and MARV. We identified 3 survivors who demonstrated a strong polyclonal serum response against all 4 filovirus GPs tested (EBOV, SUDV, BDBV, MARV), a finding that was confirmed with affinity purified GP-reactive antibodies from the polyclonal plasma of the strongest responder (subject ES 2), as well as responses against EBOV NP and VP40. These findings demonstrate that a single infection with EBOV can elicit a pan-filovirus antibody response capable of neutralizing against pathogenic variants of both ebolavirus and MARV. The data suggest that a pan-filovirus vaccine may be achievable, and that it may be possible to isolate human mAbs that neutralize all filoviruses for use in prophylaxes or therapy.

Given that there has been no documentation of multiple filovirus infection in human subjects, nor reinfection of a particular strain, the immune response generated by a subset of these Congolese survivors can be attributed to a singular infection with EBOV with high confidence. Recently, a broad pan-ebolavirus response has been observed in individuals from the 2014 West African EBOV outbreak and has shed light on the possibility of the natural generation of neutralizing antibodies against a wide range of ebolaviruses [24]. Human mAbs isolated from Ugandan BDBV or West African EBOV survivors can recognize and neutralize multiple species of EBOV (exhibiting a pan-ebolavirus pattern); however, these mAbs did not inhibit MARV [12]. The fact that a subset of these Congolese EBOV survivors possess antibodies that inhibit all pathogenic ebolavirus strains and MARV supports a model in which a truly pan-filovirus response might be induced in humans by infection or vaccination with filovirus GP antigens. The exact mechanism of action of this response requires additional experimentation.

The epitope recognized by these pan-filovirus antibodies is uncertain. We previously isolated MARV neutralizing mAbs from a MARV survivor that recognize the MARV RBS and, if the EBOV glycan cap and mucin-like domain are removed proteolytically, also bind to EBOV GP at the RBS and inhibit EBOV [25]. However, since the RBS is occluded in intact ebolavirus GP, antibodies recognizing the canonical RBS in this way are unlikely to bind and neutralize ebolavirus particles directly. Antibodies induced by ebolavirus infection that neutralize MARV have not been reported. Here, we determined that antibodies in EBOV survivor plasma competitively bound to MARV GP in areas that were known previously to be sites of vulnerability for neutralization [26]. This finding indicates that antibodies likely were generated within this particular EBOV survivor that neutralize MARV by binding to sites that are known to inhibit MARV infection. It may also be possible that this survivor generates unique mAbs that recognize different GP epitopes that individually may not neutralize in a pan-filovirus fashion but could do so in a cooperative binding mode, which has been shown for various ebolavirus species in an NHP model [27]. Collectively, these data describe a human EBOV survivor from the 2014 Boende DRC outbreak who made an unusually broad pan-filovirus neutralizing antibody response. Additional work will be required to identify if the B-cell repertoire of such individuals harbors unique mAbs that could be used for novel therapeutic options in the treatment of both ebolavirus and MARV infections. This work also may suggest the possibility of novel immune responses that may be restricted to individuals in this central African region who have been widely exposed to diverse emerging infectious diseases and viral hemorrhagic fever viruses for much longer than other areas of Africa and the world.

## Supplementary Data

Supplementary materials are available at *The Journal of Infectious Diseases* online. Consisting of data provided by the authors to benefit the reader, the posted materials are not copyedited and are the sole responsibility of the authors, so questions or comments should be addressed to the corresponding author.

Supplementary Flipped 01

Supplementary Flipped 02

Supplementary Flipped 03

Supplementary Flipped 04

Supplementary Flipped

Supplementary Figure Legend

## Notes

***Acknowledgments.*** We thank Carl W. Davis and Rafi Ahmed for their supply of critical cell reagents necessary for some experimentation conducted for this manuscript.

***Financial support.*** This project received support from the Bill & Melinda Gates Foundation (grant numbers OPP1066684, OPP1151789, and 20153705 via Atreca subcontract with the parent grant number OPP1066534 [to J. E. C. and A. W. R.]); the Faucett Catalyst Fund (to A. W. R.); the Defense Threat Reduction Agency (grant number HDTRA1-13-1-0034 to J. E. C.); and the NIH (grant numbers U19 AI109711 to J. E. C. and R21AI107420 to G. S. and contract number HHSN272201400058C to J. E. C.). The neutralization work completed at the National Institute of Allergy and Infectious Diseases (NIAID) Integrated Research Facility was supported by the NIAID Division of Intramural Research and NIAID Division of Clinical Research, and was performed by the Battelle Memorial Institute (contract number HHSN272200700016I). M. S. B. is supported by the Fogarty International Center of the NIH (award number D43TW009343) and the UCGHI.

***Potential conflicts of interest.*** All authors: No reported conflicts of interest. All authors have submitted the ICMJE Form for Disclosure of Potential Conflicts of Interest. Conflicts that the editors consider relevant to the content of the manuscript have been disclosed.
