# Supplementary figures and images for: Pan-Filovirus Serum Neutralizing Antibodies in a Subset of Congolese Ebolavirus Infection Survivors
Source: J Infect Dis. 2018 Aug 13;218(12):1929–36. doi: 10.1093/infdis/jiy453 (PMC6217721; doi:10.1093/infdis/jiy453)

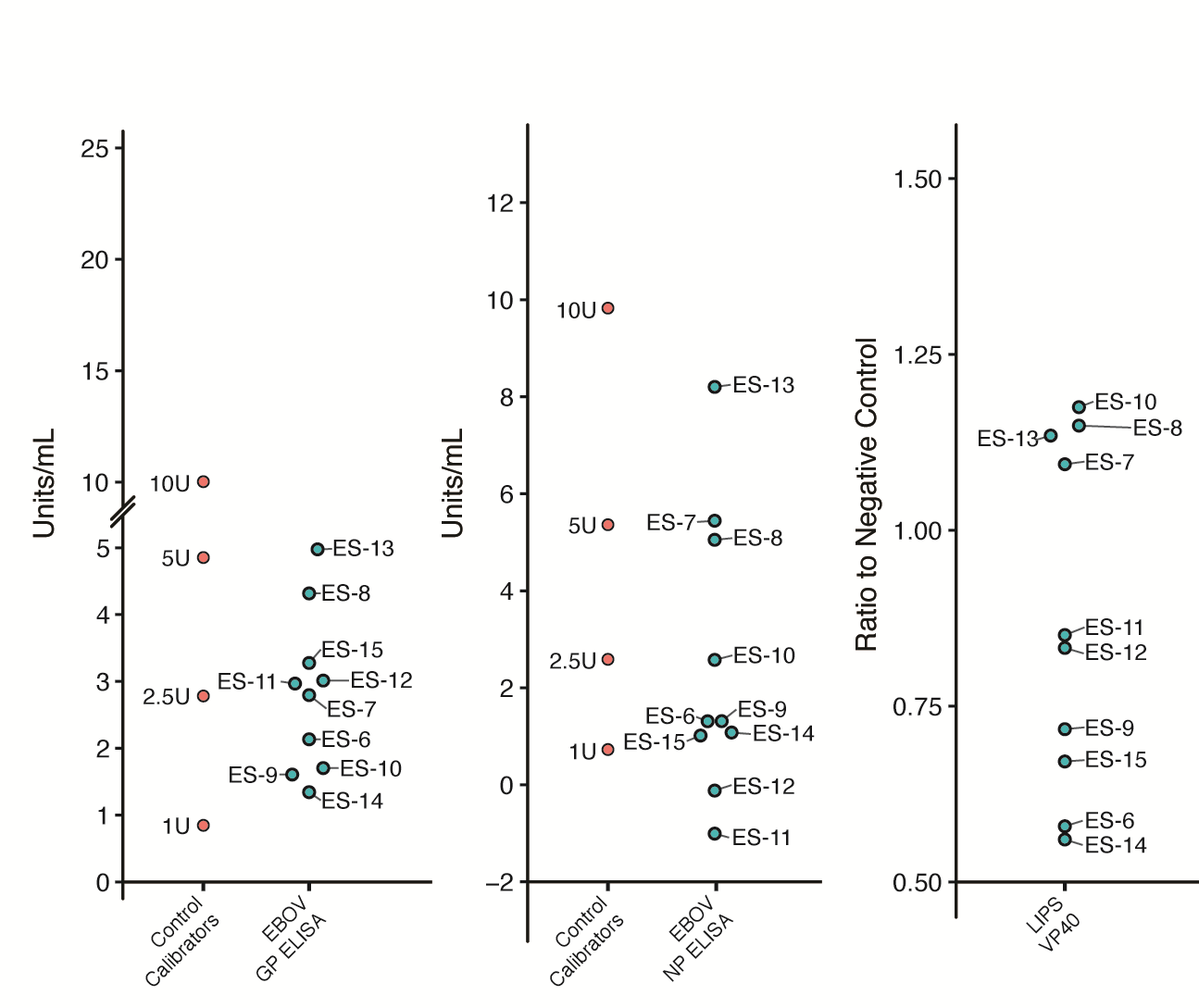

Supplement: Supplementary Flipped 01 [file jiy453_suppl_supplemental_flipped-01.png]

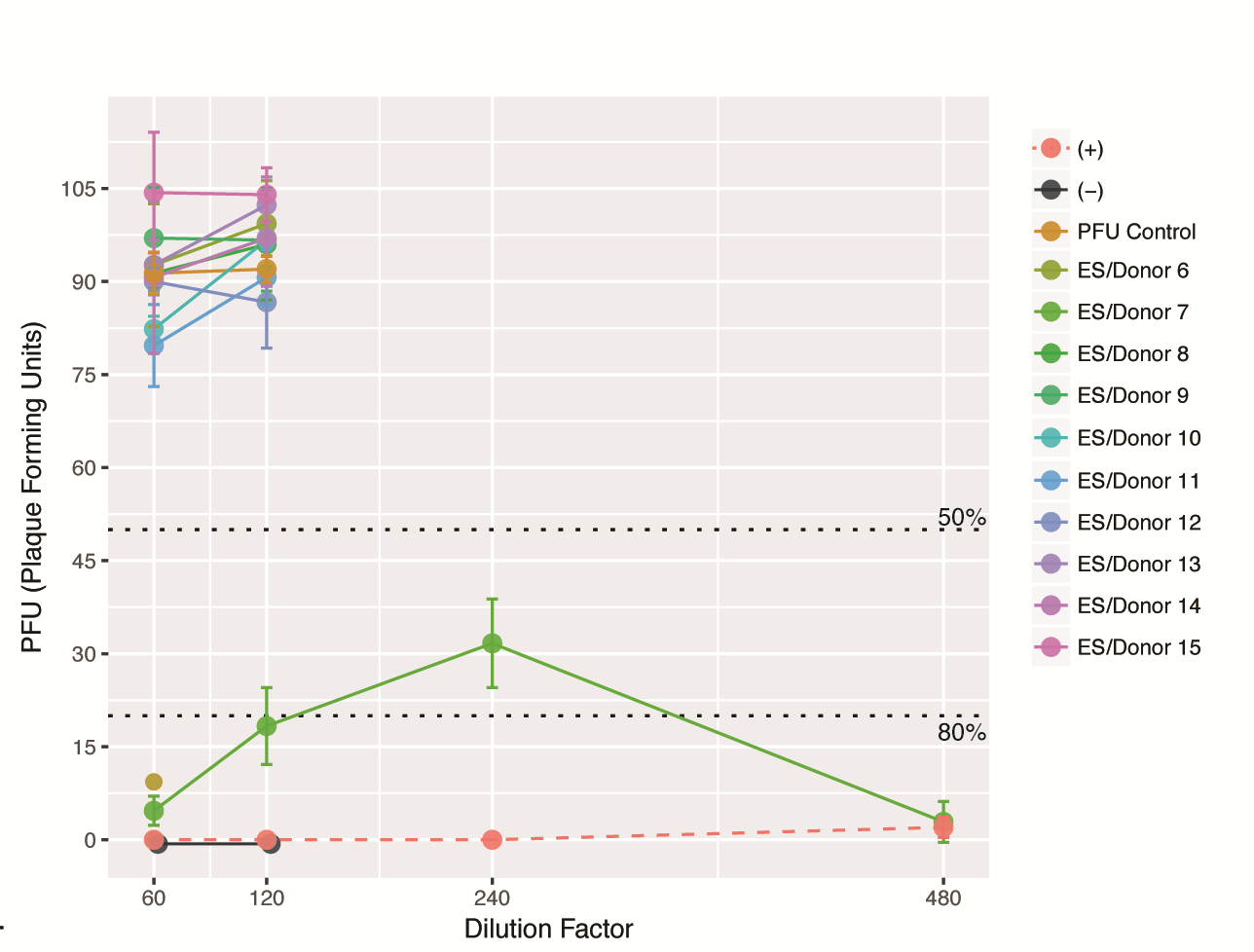

Supplement: Supplementary Flipped 02 [file jiy453_suppl_supplemental_flipped-02.png]

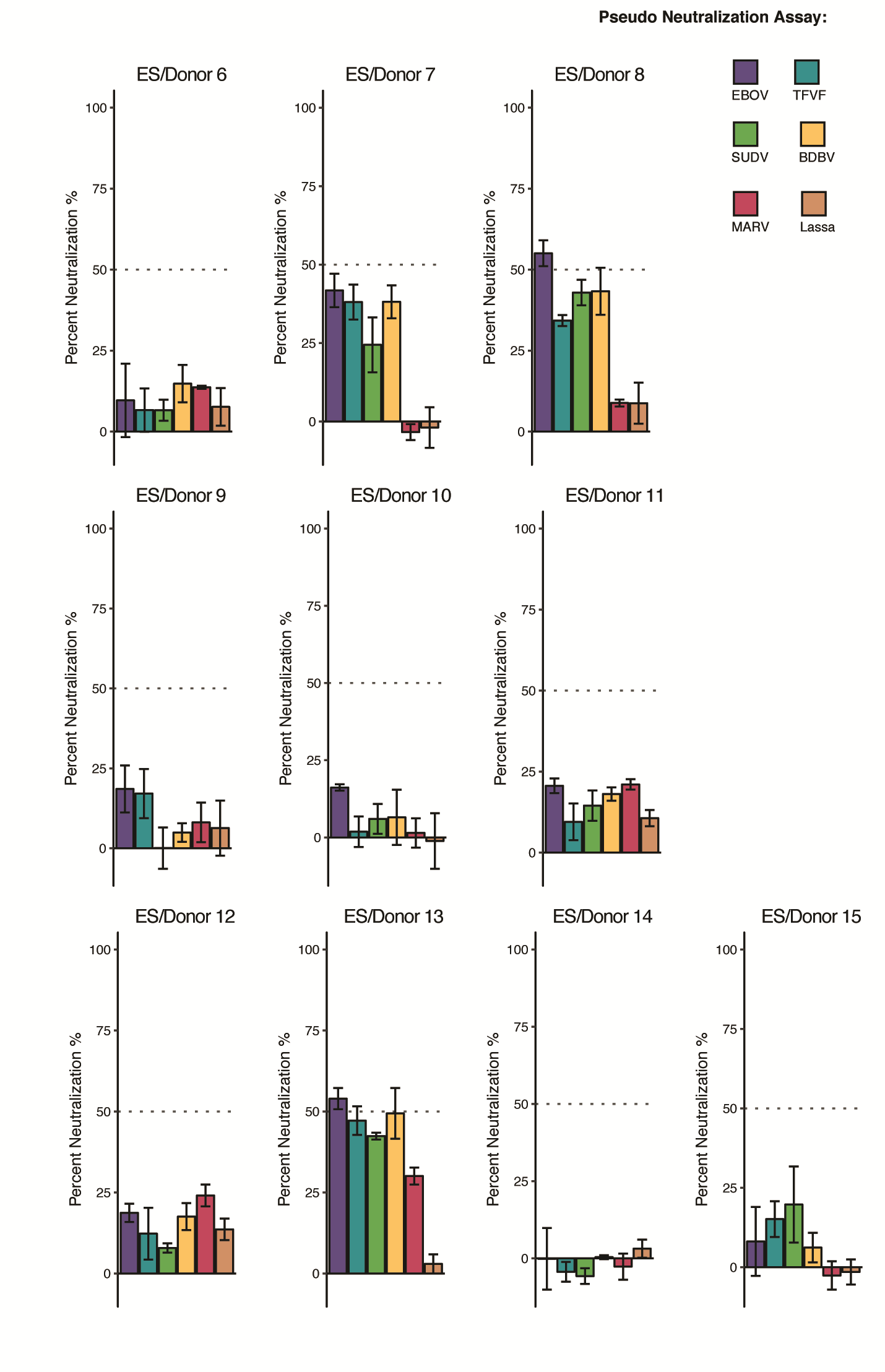

Supplement: Supplementary Flipped 03 [file jiy453_suppl_supplemental_flipped-03.png]

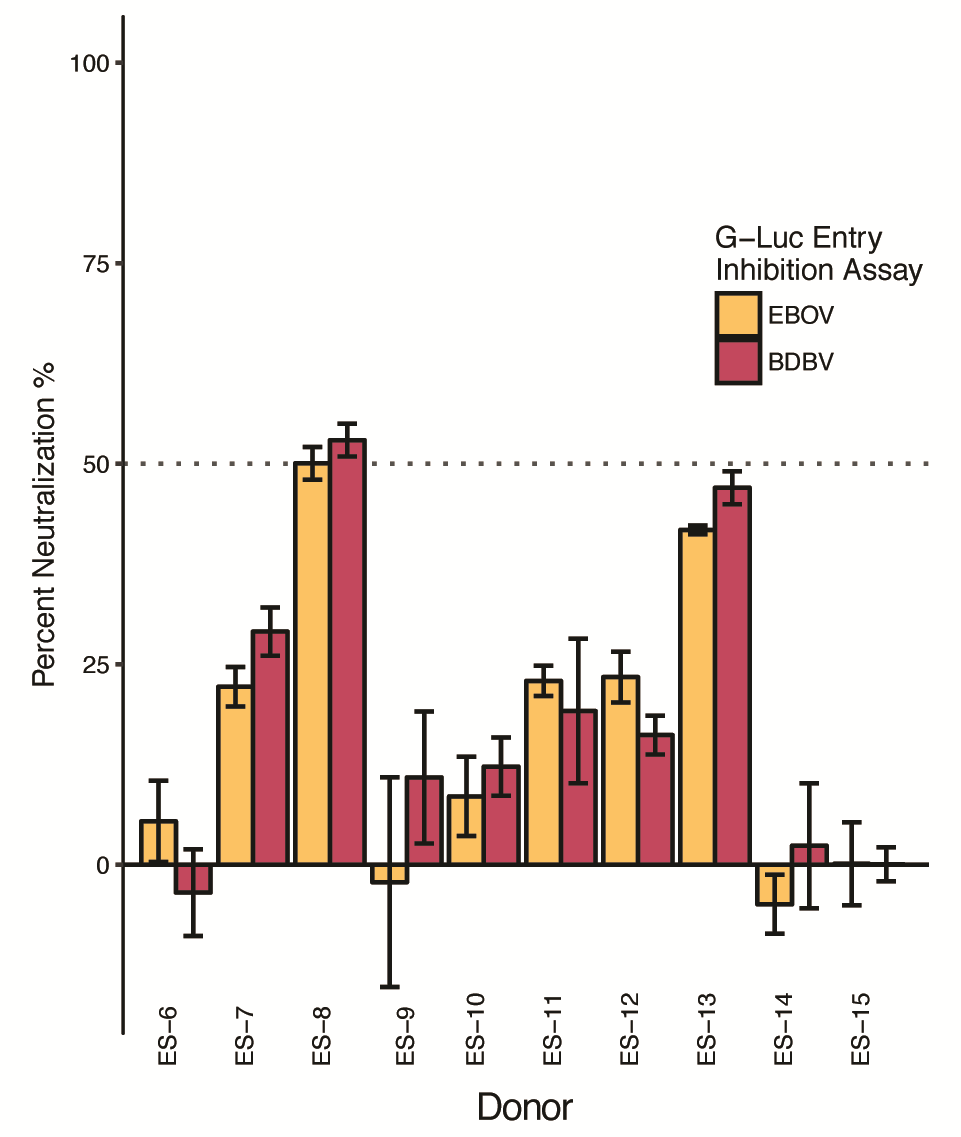

Supplement: Supplementary Flipped 04 [file jiy453_suppl_supplemental_flipped-04.png]

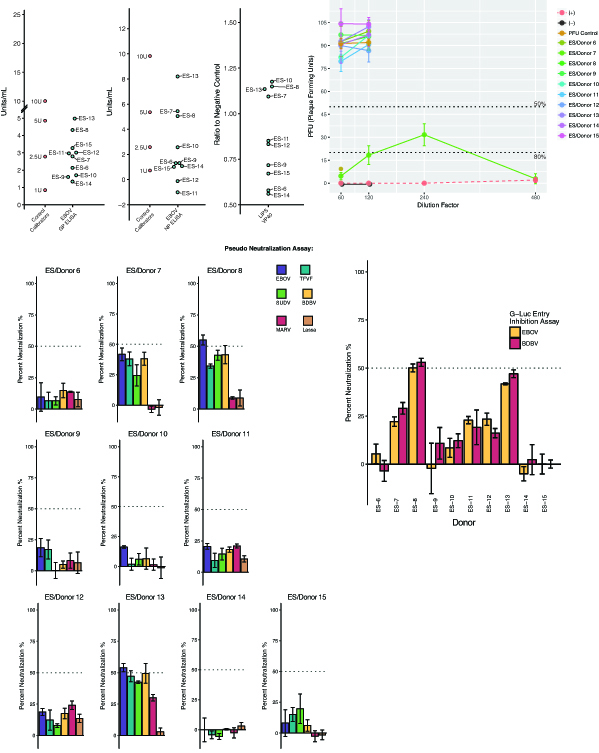

Supplement: Supplementary Flipped [file jiy453_suppl_supplemental_flipped.jpeg]
