# Supplementary material for: Pan-Filovirus Serum Neutralizing Antibodies in a Subset of Congolese Ebolavirus Infection Survivors
Source: J Infect Dis. 2018 Aug 13;218(12):1929–36. doi: 10.1093/infdis/jiy453 (PMC6217721; doi:10.1093/infdis/jiy453)
Supplement: Supplementary Figure Legend [file jiy453_suppl_supplementary_figure_legend.docx]

**Supplementary figure legend:**

**Supplemental Figure 1**.

**A)** Serological assessment of the 10 survivors that did not demonstrate confident pan-ebola/filovirus neutralization activity. **B)** VSV pseudovirus neutralization assays for the 10 additional survivors in the study cohort. **C)** PRNT assessment of the 10 survivors that did not demonstrate pan-filovirus neutralization activity. **D)** G-luc inhibition assay for EBOV and BDBV using serum from those survivors who did not respond in a pan-filovirus fashion. Concentrations and statistics correspond to those figures in main text.
